# Functionalized Silk Fibroin and Mucin Hybrid Material for Targeted EGF and Papain Delivery in Wound Healing

**DOI:** 10.1021/acsomega.5c03320

**Published:** 2025-08-13

**Authors:** Fernando José Soares Barros, Laise Maia Lopes, Sedef Ilk, Rodrigo Silveira Vieira, Thomas Crouzier, Mariana Agostini de Moraes, Marisa Masumi Beppu

**Affiliations:** † KTH Royal Institute of Technology, Department of Chemistry, Division of Glycoscience, SE-10044 Stockholm, Stockholm County, Sweden; ‡ 28132 State University of Campinas (UNICAMP), School of Chemical Engineering, Department of Materials and Bioprocess Engineering, Av Albert Einstein, 500, CEP 13083-852 Campinas, SP, Brazil; § Niğde Ömer Halisdemir University, Faculty of Medicine, Department of Immunology, TR-51240 Niğde, Turkey; ∥ Federal University of Ceará (UFC), Department of Chemical Engineering, Campus do Pici, Bloco 709, CEP 60440-900 Fortaleza, CE, Brazil; ⊥ Technical University of Denmark (DTU), Department of Health Technology, Ørsteds Plads, Building 345C, DK-2800 Lyngby, Denmark; # Federal University of São Paulo (UNIFESP), Department of Chemical Engineering, Rua São Nicolau, 210, CEP 09913-030 Diadema, SP, Brazil

## Abstract

Silk fibroin (SF) and mucin are extensively recognized as promising biomaterials for wound dressings due to their outstanding biocompatibility, biodegradability, and ability to support cell growth and tissue regeneration. In this study, we developed a hybrid SF/mucin wound dressing (HYB) using tetrazine and norbornene click chemistry to enhance its structural and functional properties. The robust assembly resulted in a dual-phase material with a dense SF membrane and a porous mucin hydrogel (MH). Scanning electron microscopy confirmed the successful integration and tight adhesion between these polymers. The hybrid material exhibited a controlled release of bioactive agents, with epidermal growth factor (EGF) showing a sustained release of up to 48% over 48 h. The optimized 25 mg/mL mucin hydrogel showed efficient EGF release and performance comparable to higher concentrations. It was selected for papain loading to reduce material usage without compromising efficacy. HYB showed a higher papain release rate of 36% compared to the bare SF membrane. Additionally, the hybrid material exhibited enhanced mechanical strength, optimized water vapor permeability comparable to commercial wound dressings, and improved cell proliferation relative to its individual components. Cytotoxicity assays demonstrated that the papain-loaded hybrid material is a viable candidate for wound dressing applications. These results suggest that the click-chemistry-functionalized SF/mucin hybrid material holds significant potential as an advanced wound dressing, capable of promoting tissue regeneration while maintaining a moist environment conducive to healing.

## Graphical abstract

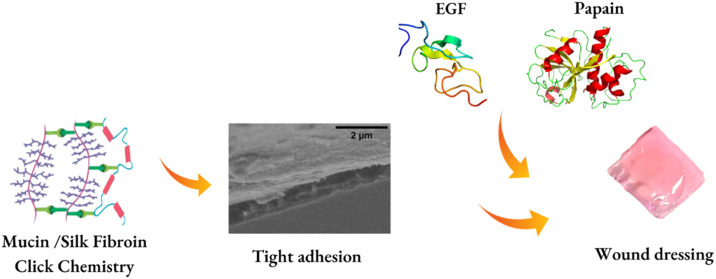

## 1 Introduction

Biomaterials have become essential components in developing strategies to promote the dynamic and complex tissue regeneration process during wound healing.^1^ Wound dressing materials not only create a closed environment to protect the wound from dehydration and infections but also provide biochemical and physical cues to stimulate cellular processes.^2^ Natural polymers in wound dressing are particularly attractive due to their biocompatibility, biodegradability, intrinsic bioactivity, and sustainability.^3^ In this work, we explore the potential of silk fibroin protein and mucin glycoprotein as key building blocks of wound dressing materials.

Silk fibroin (SF) is a fibrous protein from the silkworm (*Bombyx mori*) that has a long-standing history of use in surgical tissues and sutures.^4^ The intrinsic properties of silk fibroin render it highly suitable for biomedical applications because it is also nontoxic, nonimmunogenic, and highly biocompatible with a wide range of animal species.^5^ In addition, fibroin membranes can be formed into flexible dressings that can adhere to the wound and prevent excessive flow of exudates, proteins, and substances that promote cell proliferation.^6^ Previous studies have applied fibroin membranes, sponges, and scaffolds, either in pure form^7^ or as blends with other biopolymers, such as chitosan,^8^ alginate,^9^ collagen,^10^ and keratin,^11^ in applications as wound dressing both *in vitro* and *in vivo*. These studies demonstrated excellent exudate absorption, faster healing, increased re-epithelialization, and reduced wound inflammation.^6^ However, silk fibroin membranes lack the enhanced bioactivity exhibited by other materials, which could accelerate wound healing. Combining silk fibroin with other biomaterials to form a hybrid structure could address this limitation. Mucins are a family of large glycoprotein polymers that exist both as membrane-tethered molecules and as major components of mucus gels secreted by goblet cells in the epithelium.^12^ Mucins exhibit hydration, lubrication, and barrier properties. Mucins are also bioactive, with potent immunoregulatory activities, dampening the activation of macrophages *in vitro* and the foreign body reaction *in vivo*, achieved by binding and activating cell surface receptors and interacting with bioactive proteins and peptides.^13–15^ Mucins likely contribute to wound healing in mucosal surfaces and skin, as evidenced by the well-documented effects of animal wound licking, which combines mucins from saliva with other bioactive proteins to accelerate healing. The bioactivity of mucins has been observed in both three-dimensional gels and mucin-immobilized thin films.^16^ Hydrogels offer a favorable environment for enhancing cell adhesion, growth, proliferation, and differentiation—key processes that contribute to accelerated wound healing within a shorter time frame.^17^ Hydrogel scaffolds can accommodate cells involved in tissue regeneration, providing them with a three-dimensional (3D) water-containing network structure similar to the natural extracellular matrix.^18^ Robust mucin hydrogels (MHs) can be engineered by cross-linking mucins to each other or by blending with mucoadhesive molecules, which could enable their incorporation in wound dressing materials.^13,14^ In recent works, synthetic mucin hydrogels were able to safeguard intervertebral discs from degeneration after discectomy.^19^

To enhance the performance of SF, functionalization is often necessary, particularly to facilitate blending with other polymers and improve chemical compatibility. This approach has been widely used to enhance the mechanical strength and stability of SF materials in diverse biomedical contexts.^20–22^ Common functionalization approaches for silk fibroin often involve harsh conditions or low specificity, risking structural damage and reduced biocompatibility. Click chemistry, in particular, presents a significant advantage with its mild, efficient, and high-yielding reactions, minimizing byproducts and harsh conditions while preserving fibroin properties and enabling versatile modifications. This methodology enables precise and reliable functionalization of SF under aqueous conditions, expanding its versatility as a substrate for drug delivery, enzyme attachment, and cell interaction, without compromising its biocompatibility or mechanical integrity. In this regard, we propose the use of click chemistry to functionalize SF and mucin in order to enable specific interactions between these two proteins at the interface of the hybrid material and, at the same time, preserve the intrinsic functional and biological properties of them.^22^

In addition to the polymer matrix of the wound dressing, incorporating active agents is a key strategy to accelerate healing. The latex of papaya (*Carica papaya*) has a long tradition of use in wound healing and treating burns, with reports of usage by indigenous tribes in South America and Africa.^23^ Papaya latex contains a complex mixture of cysteine endopeptidases, including papain, a proteolytic enzyme known for its role in chemical debridement of tissues. Papain also exhibits bactericidal, bacteriostatic, and anti-inflammatory effects on wounds.^23–25^ The epidermal growth factor (EGF) is also widely used in wound treatments. EGF is a polypeptide composed of 53 amino acids that promotes dermal regeneration by stimulating cellular migration, proliferation, and angiogenesis, essential for successful wound healing and tissue repair. However, EGF has certain limitations, including reduced stability at room temperature, especially in terms of biological activity.^26^ Therefore, stabilizing EGF can improve its capacity to accelerate wound healing.^27,28^

Here, we hypothesize that combining silk fibroin, mucins, and a bioactive compound, such as papain or EGF, into a hybrid material would yield a system with optimal physical and biological properties for wound healing. The silk fibroin membrane serves as a robust physical scaffold for the mucin hydrogel, while the mucin and papain or EGF provide complementary bioactivities, stimulating cell proliferation and immune regulation at the wound site.

## 2 Experimental Procedure

### 2.1 Materials

Silkworm cocoons from *Bombyx mori* were supplied by Bratac, Brazil. Papain was purchased from Dinâmica, Brazil. Amine derivatives of tetrazine (Tz) and norbornene (Nb) were purchased from Click Chemistry Tools and TCI Europe N.V., respectively. Mucin, extracted from bovine submaxillary (BSM) glands, and all other chemicals were purchased from Sigma-Aldrich, Sweden.

### 2.2 Silk Fibroin (SF) Solution

The silk fibroin aqueous solution was prepared using silkworm cocoons from *B. mori*, adapting the method described elsewhere.^29^ Silkworm cocoons were degummed using 1 g·L^–1^ Na_2_CO_3_ solution at 85 °C for 30 min to remove sericin. The process was repeated three times, and then, the cocoons were washed with deionized water. The silk fibers were dried at room temperature and dissolved in a CaCl_2_:CH_3_CH_2_OH:H_2_O (1:2:8 molar ratio) solution at 85 °C to a concentration of 10 wt %. To remove the salts, the solution was dialyzed with ultrapure water for 3 days at 10 °C using a dialysis membrane (MWCO 3.5 kDa—Thermo Fisher Scientific, EUA), and the water was changed every 24 h for 3 days. The solution was centrifuged (2300 RCF, 30 min) to remove insoluble particles. Then, a 2.7% (w/v) aqueous fibroin solution was obtained, with its content determined gravimetrically by drying the solution in a Petri dish.

### 2.3 SF Functionalization

After quantification, the 2.7% SF (w/v) aqueous solution was functionalized with tetrazine groups. *N*-hydroxysuccinimide (NHS) and 1-ethyl-3-(3-dimethylaminopropyl) carbodiimide hydrochloride (EDS) were added to the silk fibroin solution at 10% (w/v) and 20% (w/v) of the fibroin weight in solution, respectively, using a method adapting previous works on SF functionalization^22^ and tetrazine click chemistry.^30^ Subsequently, 1 mmol of tetrazine per gram of silk fibroin was added to the mixture. The system was kept without stirring at 4 °C for 2 h. The solution was dialyzed with ultrapure water at 10 °C using a dialysis membrane (MWCO 3.5 kDa—Thermo Fisher Scientific, EUA), and the water was changed every 24 h for 3 days.

### 2.4 SF Membrane

Membranes were prepared for both the SF aqueous solution and the tetrazine-functionalized SF aqueous solution (SF-Tz), both at 2.7% (w/v). The membranes were made by placing each solution in a Petri dish and casting at room temperature. After water evaporation, the membranes were cross-linked through water vapor annealing.

### 2.5 Mucin Hydrogel (MHs)

The mucin hydrogel was prepared using mucin extracted from bovine submaxillary (BSM) glands, which was dissolved in a solution of 0.1 mol/L MES buffer and 0.3 mol/L NaCl, pH 6.5, at 4 °C for 24 h with vigorous agitation, according to the method described elsewhere.^30^ The mucin was functionalized with tetrazine (BSM-Tz) and norbornene (BSM-Nb) groups, following a process similar to that used for silk fibroin. NHS and EDC were added at 4 mmol per gram of mucin in different beakers. In one beaker, tetrazine-amine was added at a rate of 1 mmol of tetrazine per gram of mucin; in the other, 5-norbornene-2-methylamine was added at 2 mmol per gram of mucin. Both beakers were kept under vigorous agitation overnight at 4 °C. After the reaction, the solutions were dialyzed using float-a-lyzer 100 kDa dialysis tubes (Sigma-Aldrich) at 4 °C for 3 days, with three daily changes. On the first 2 days, a solution of 0.3 mol/L NaCl was used, and ultrapure water was used on the final day. After dialysis, the mucin was lyophilized for 48 h. To form the hydrogels, mucin functionalized with tetrazine and norbornene was dissolved in phosphate-buffered saline (PBS) in different tubes at 2.5% (w/v) and 5% (w/v) and mixed in equal volumes. Complete hydrogel formation occurred within 1 h.

### 2.6 Hybrid Material (HYB) Preparation

To form the hybrid material, the SF-Tz membrane was cut into circular shapes of 6 mm in diameter. Mucin solutions functionalized with tetrazine and norbornene were mixed and immediately placed on the membrane. The system was left to rest for 1 h to allow complete hydrogel formation.

### 2.7 Characterization

The materials were characterized in terms of chemical composition using Fourier transform infrared spectroscopy with attenuated reflectance accessory (FTIR-ATR), morphology through scanning electron microscopy (SEM), and water vapor permeability.

SEM (S-4800, Hitachi, Japan) was used to characterize mucin hydrogels, SF membranes, and hybrid materials. The mucin hydrogels and the hybrid materials were prepared by the critical point drying method using ethanol to dehydrate the samples and preserve the hydrogel structure. The samples were fractured and coated with a gold layer before analysis. The average pore diameter was calculated using ImageJ software for 50 pores.

FTIR-ATR analysis was performed using an infrared spectrometer with Fourier transform (Thermo Scientific, model Nicolet 6700) with ATR accessory (Thermo Scientific, model Nicolet Continuum). The range was 4000–675 cm^–1^, with a resolution of 4 cm^–1^ and 64 scan accumulation.

Water vapor permeability was determined following the ASTM E 96/E 96 M standard method. An apparatus consisting of a 30 mL acrylic container with an area of 15.2 cm^2^ (cell 1) containing anhydrous calcium chloride was used to hold the silk fibroin membrane. Cell 1 was then placed inside a 500 mL acrylic container (cell 2) with a saturated NaCl solution to maintain 75% relative humidity. Cell 1 was weighed every 12 h for 5 days, and the increase in mass was used to calculate the water vapor permeability rate of the membrane. At the end of the test, membrane thickness was measured using a Mitutoyo MDC-25S digital micrometer.

### 2.8 Epidermal Growth Factor (EGF) and Papain Incorporation

EGF was incorporated into the different matrices to analyze its release profile from the studied materials: SF membranes, functionalized SF membranes, MHs at concentrations of 25 and 50 mg/mL, and HYB membranes composed of functionalized SF and MH.

In both SF and functionalized SF membranes, EGF was incorporated as follows: after fibroin dialysis, an EGF solution was added to achieve a final concentration of 100 ng/mL, while the final concentration of silk fibroin was set to 10 mg/mL. The membranes were prepared in 96-well plates, with 124 μL of EGF-containing fibroin solution added to each well. Membranes were formed *via* casting, and cross-linking was performed by annealing, as previously described.

In the case of MH, the EGF solution was mixed with mucin functionalized with tetrazine or norbornene prior to mixing and hydrogel formation. The final concentration of EGF in the hydrogels was 100 ng/mL. After EGF incorporation, the SF-Tz membranes and 25 mg/mL MHs were characterized by FTIR.

For the HYB, EGF was incorporated in the same manner as in the functionalized SF membranes using the same concentrations. After membrane formation and cross-linking, the hydrogel was placed on top of the functionalized silk fibroin membrane, forming the hybrid membrane.

Papain immobilization on the functionalized SF membrane and for HYB was achieved *via* physical adsorption. Tetrazine-functionalized SF membranes (6 mm in diameter) were placed in individual wells of a well plate. A papain solution (5 mg/mL in phosphate-buffered saline, PBS) was then added on top of each membrane to fully cover the surface. The membranes were incubated overnight at 4 °C to allow for papain adsorption into the Tz-SF matrix.

For the HYB material, Tz-functionalized silk fibroin membranes (6 mm in diameter) were also used as the base. Prior to papain loading, a mucin hydrogel layer was formed by mixing equal volumes of mucin–tetrazine (Muc/Tz) and mucin–norbornene (Muc/Nb) solutions, and this mixture was immediately applied on top of the Tz-SF membranes. Once the hydrogel layer was formed, the papain solution (5 mg/mL in PBS) was added on top of the mucin-coated membranes. The membranes were incubated overnight at 4 °C to allow for enzyme diffusion and interaction with the composite structure.

### 2.9 Epidermal Growth Factor (EGF) and Papain Release

EGF release was assessed by incubating samples with 200 μL of PBS buffer (pH 7.4) at 37 °C for 7 days. Before the release assay, samples were washed twice with 200 μL of PBS (pH 7.4) for 1 min, and this wash was considered time zero. The EGF concentration was quantified using a commercial ELISA kit (Thermo Fisher, Sweden). Samples were added to wells precoated with anti-EGF antibodies and incubated to allow binding. After washing, a biotinylated detection antibody and HRP-conjugated streptavidin were added. Following incubation, the TMB substrate was added for color development. The reaction was stopped with sulfuric acid, and absorbance was read at 450 nm. The EGF concentration was calculated using a standard curve generated from known EGF standards. The tests were performed in triplicate.

Papain release from the hybrid material was analyzed using a dialysis method in a medium of PBS at 37 °C for 6 h. The SF-Tz and HYB was added to dialysis bags (Mw: 12000–14 000 Da) and stirred at 150 rpm in release media at 37 °C. Aliquots (3 mL) were withdrawn at predetermined intervals, replaced with fresh medium, and analyzed by ultraviolet–visible (UV–vis) spectroscopy at 278 nm. Each test was performed in triplicate.

The release kinetics were modeled using the Korsmeyer–Peppas equation (eq 1).

1 $$\frac{M_{t}}{M_{\infty }}=k\cdot t^{n}$$

where *M_t_*/*M*_∞_ is the drug release fraction, *t* is the variation of time in the release, *k* is the kinetic constant (min^–*n*^), and *n* is the diffusion constant, which depends on the release mechanism.^31^

### 2.10 Cell Culture

HaCat keratinocyte cells were cultured in DMEM/F-12 (1:1) (1x) + Glutamax media with 10% fetal bovine serum and 1% streptomycin and penicillin. Cells were maintained at 37 °C in a 5% CO_2_ atmosphere, with media changed every 4 days and subcultured using trypsin.

### 2.11 Cell Viability

Cell viabilities of the SF membrane, hybrid material, and papain-loaded HYB were assessed using the AlamarBlue assay (Thermo Fisher Scientific). Quintuplicate samples of materials sterilized with UV radiation were prepared using a 6 mm biopsy punch. Cells (10 μL per well) were seeded into 96-well plates and incubated for 3 h. After incubation, 10 μL of AlamarBlue was added to each well and incubated for 1 h. Fluorescence intensity was measured using a ClarioStar plate reader (BMG Labtech) with excitation at 560 nm and emission at 590 nm. Controls included cells exposed to supplemented DMEM (negative control) and ethanol (positive control).

Statistical analyses were performed using GraphPad Prism 8.0.2 to compare the samples SF, HYB, HYB + Pap, and controls. Brown–Forsythe and Bartlett’s tests were used to assess the homogeneity of variances, and the Shapiro–Wilk test was applied to assess the normality of the data. One-way ANOVA followed by Tukey’s multiple comparisons test was applied to determine statistically significant differences among group means. Differences were considered significant at *P* < 0.05.

## 3 Results and Discussion

### 3.1 Hybrid Material (HYB) Assembly

We first aimed to physically assemble the two materials to combine the material properties of silk fibroin films and mucin hydrogels. Mucin hydrogels applied to unmodified silk fibroin films exhibited no adhesion. The click chemistry between norbornene (Nb) and tetrazine (Tz) was chosen as a strategy to assemble the hybrid material due to advantages such as high yields, stability of norbornene in solution, and no known reactivity with naturally occurring molecules.^30^ In an attempt to form more stable bonds between the materials, we functionalized silk fibroin with tetrazine, forming an SF membrane. The mucin hydrogel is obtained by first functionalizing the mucin molecules with either Tz and Nb groups, producing covalent bonds when mixed in solution, through an inverse electron demand Diels–Alder cycloaddition reaction.^30^ For the mucin hydrogel, the EDC/NHS coupling was used to localize the cross-links on the mucin core (protein–protein cross-linking or Prot–Prot), Figure 1.^13^ We hypothesize that a possible mismatch between Tz and Nb groups in the mucin gel would leave an excess Norbornene available to interact with the tetrazine-functionalized membrane (Figure 1).

**Figure 1:**
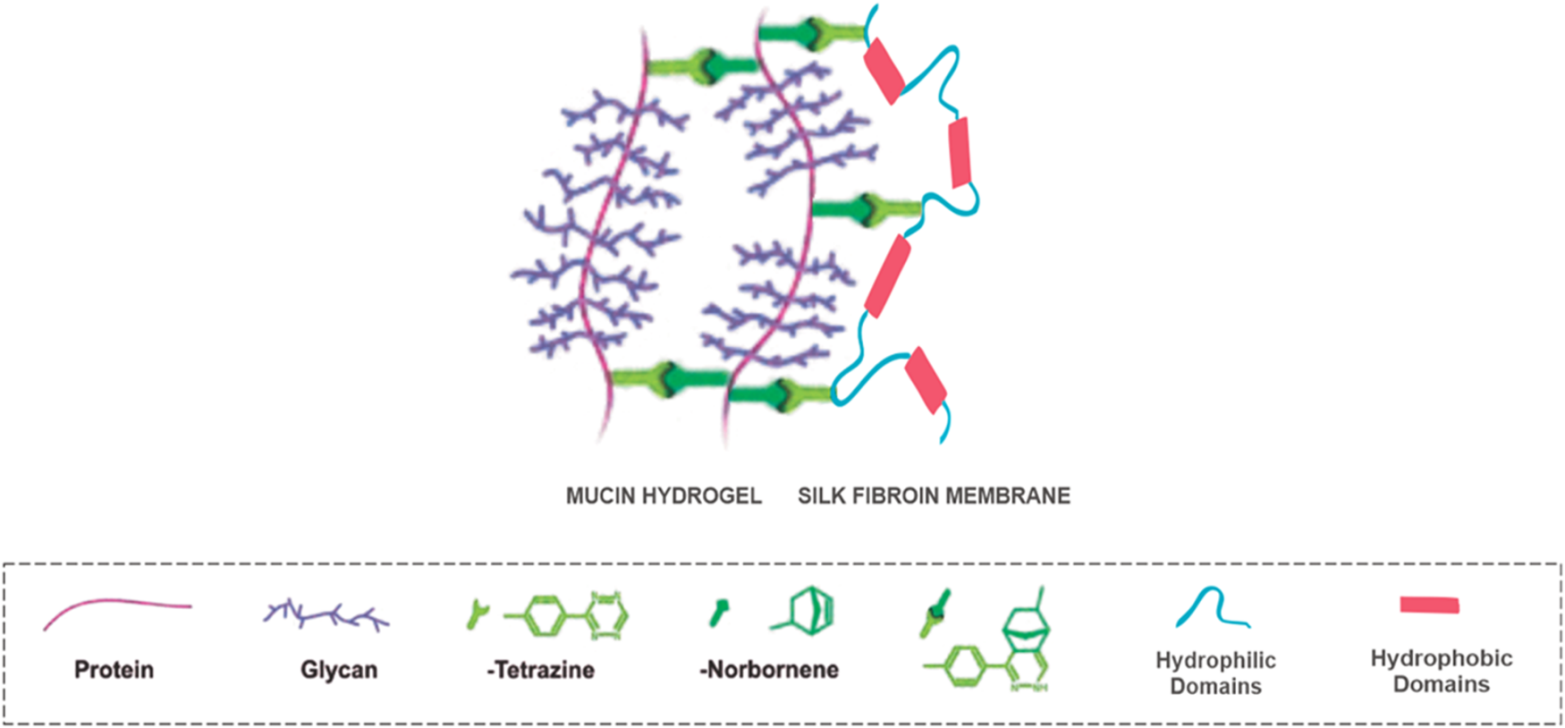
Click chemistry involved in forming the SF-Tz/mucin hybrid.

The tetrazine-functionalized silk fibroin membrane had a pink color as opposed to the colorless nonfunctionalized membranes (Figure 2A). Upon assembly with MH, the two components formed a cohesive hybrid structure that could be shaped into various geometries, such as round or square forms (Figure 2B). The mucin hydrogel exhibited a light pink, semitransparent appearance, which contributed to the overall soft pink and translucent visual characteristic of the hybrid material.

**Figure 2:**
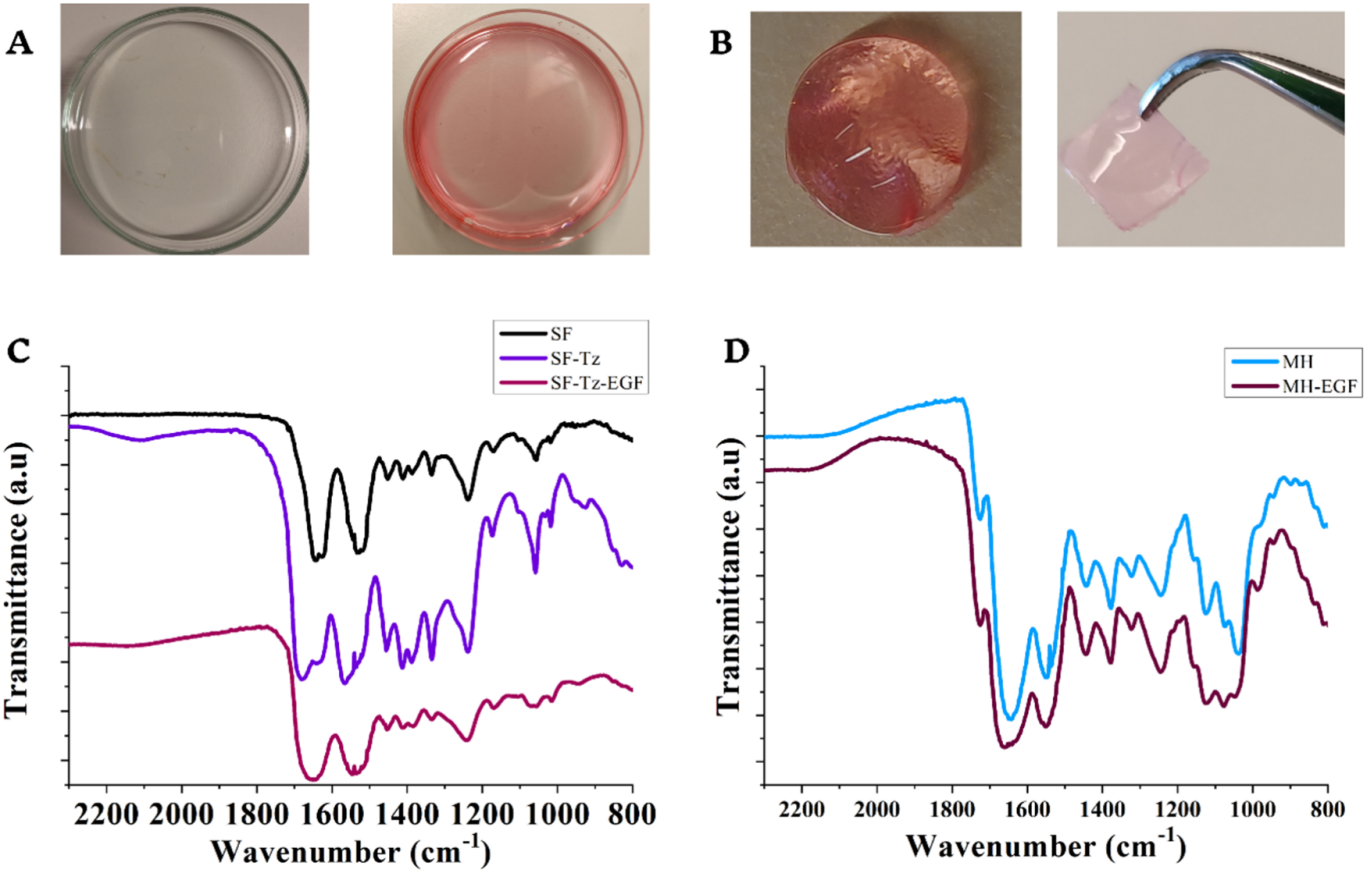
(A) SF membranes (left) before and (right) after functionalization with tetrazine. (B) Hybrid material of the silk fibroin membrane and mucin hydrogel on the shapes: (left) round and (right) square. (C) Infrared spectrum (FTIR-ATR) of the silk fibroin membrane, SF-Tz membrane, and SF-Tz after EGF loading. (D) Infrared spectrum (FTIR-ATR) of the MH and MH after EGF loading.

We confirmed the presence of the characteristic bands of the functional groups of SF and mucin by infrared spectroscopy (FTIR-ATR) of the SF membrane, SF-Tz membrane, and MH 25 mg/mL, as presented in Figure 2. A summary of the main bands observed for each material is shown in Table S1.^32^ The main peaks for the SF membrane were 1643 and 1626 cm^–1^ for amide I in the β-sheet conformation, 1532 cm^–1^ for amide II in the α-helix conformation, and 1238 cm^–1^ for amide III in the β-sheet conformation, Figure 2C.^10,33^ The cross-linking of the SF membrane was performed using the annealing method at 25 °C. According to previous studies, this method is effective in stabilizing the membranes; however, at the temperature applied, part of the structure remains in the α-helix conformation even after cross-linking, resulting in a crystallinity degree of approximately 30%.^34^ After functionalization of SF with Tz, peaks shifts in the infrared spectrum were observed for the SF-Tz membrane for amide I (1681 and 1630 cm^–1^) and amide II (1566 cm^–1^). The peak near to 2100 cm^–1^ is related to the aromatic ring present in the tetrazine structure. These spectral shifts may indicate the chemical region where SF-Tz bonding occurs. Following EGF loading, the SF-Tz membranes exhibited further spectral shifts, with peaks observed at 1651 cm^–1^ (amide I), 1547 and 1536 cm^–1^ (amide II), and 1242 cm^–1^ (amide III), suggesting that EGF incorporation modifies the conformation of amides I and II to a random coil structure.^10,33^

The main peaks for MH 25 mg/mL were observed at 1726, 1643, 1544, 1443, 1245, and 1038 cm^–1^, corresponding to carboxyl groups from sialic acid, amide I, amide II, C–H_2_ vibration, amide III, and carbohydrate/sugar region, respectively.^35–37^ After EGF loading, peaks shifts were observed for amide I (1662 cm^–1^) and amide II (1551 cm^–1^), Figure 2D. It was not possible to obtain the FTIR spectrum for the SF-Tz/MH interface due to the difficulty in precisely locating the interfacial region. However, a previous study on the SF–mucin blend suggests that, when previously mixed in solution, the polymers may interact through the amine groups of SF and carboxyl and hydroxyl groups of mucin.^38^

The successful assembly of our SF/mucin material using click chemistry underscores the effectiveness of this approach for functionalizing silk fibroin. In previous works, tetrazine/norbornene were used in SF biomaterials. For instance, silk fibroin (SF) microgel-embedded poly­(ethylene glycol) (PEG) hydrogels were fabricated by dual-mode cross-linking based on thiol–ene photoclick chemistry and β-sheet formation of SF. Nb-functionalized SF was incorporated into PEG hydrogels by photo-cross-linking. The approach enables rapid and selective cross-linking, enhancing the hydrogel mechanical properties and biofunctionality while maintaining biocompatibility.^39^ A collagen–silk fibroin hydrogel was engineered using Tz and Nb click chemistry. This method enabled fast gelation, tunable mechanical properties, and improved cell compatibility, making it suitable for tissue engineering.^40^

### 3.2 Scanning Electron Microscopy (SEM)

Scanning electron microscopy (SEM) was used to evaluate the surfaces of the components of the hybrid material. Figure 3A,B presents the surface and cross section images of the SF membrane. The SF membrane appeared smooth and homogeneous, without fibrils. This is a typical feature of the SF membrane reported in other studies in the literature.^29^ The hybrid material, Figure 3C,D, shows two distinct phases, a dense one formed by SF and a porous one formed by the MH. It is possible to notice that the interaction between the materials occurs only on the surface without interpenetration. This finding suggests that the bonds were strong at the interface and blended to form a cohesive material.

**Figure 3:**
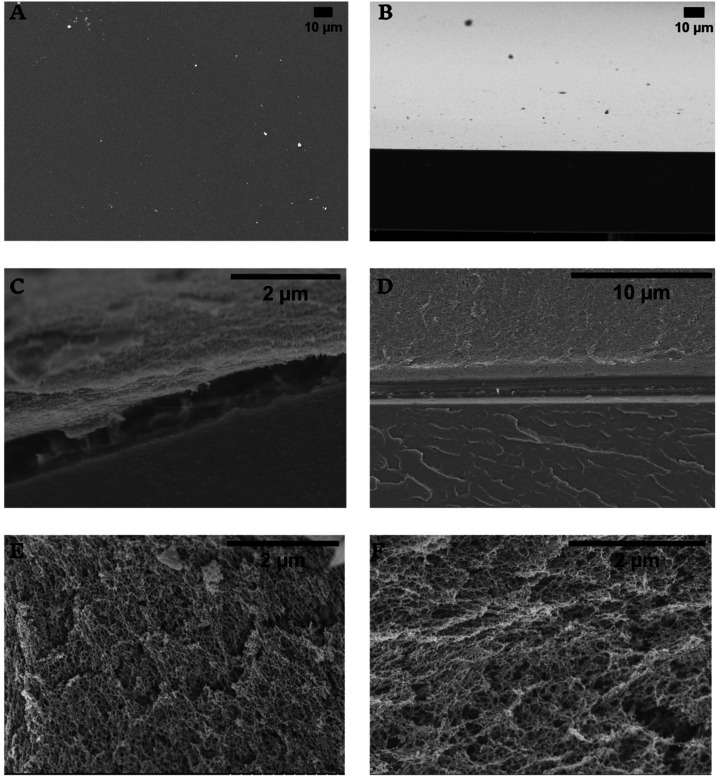
Micrographs of the silk membrane: (A) surface and (B) cross section. Cross sections of the SF and mucin hydrogel hybrid material with 2.5% (w/v) gel (C) and 5% (w/v) gel, and (D) upper layer is the mucin gel and the bottom layer is the SF membrane. Cross sections of (E) 2.5% and (F) 5% MH.

The presence of fibrils in silk fibroin in the bulk of the membrane (Figure 3C) can be explained by the method of sample preparation for SEM. The hybrid material and the mucin gel were dried by the critical point method to preserve the porous structure and obtain an image more representative of the morphology; however, the ethanol used in this process could affect the silk fibroin membrane. Mucin gels have highly interconnected nanometric porous, which appear slightly larger in 2.5% than in the 5% gel, Figure 3E,F. SEM images have confirmed that the two materials have different structures. One is dense and smooth, and the other is rougher, fibrillated, and less dense. This should lead to different mechanical properties desired for applying this hybrid material as a wound dressing. A tough silk support makes it proper to be held by the patient, and the hydrogel brings a soft interface with the wound, preventing discomfort and pain. For instance, a bilayered dressing composed of a wax-coated silk fibroin fabric as the fibroin/gelatin as the bioactive layer has shown enhanced wound healing properties. The dense layer provided mechanical strength and prevented adhesion to the wound, while the porous layer supported cell proliferation and tissue regeneration.^41^ A further demonstration of this dual-functionality design is provided by a sandwich-like composite dressing integrating a hydrophobic silk fibroin membrane, a superabsorbent chitosan-konjac glucomannan sponge, and a hydrophilic cellulose acetate membrane infused with graphene oxide. This structure facilitated continuous exudate drainage and maintains optimal wound moisture, with the dense silk fibroin layer offering protection against external contaminants.^42^ Additionally, a hybrid membrane formed by hot pressing flat silk cocoons with carboxymethyl chitosan has been developed to mimic the structure of skin. This composite exhibited excellent mechanical properties and biocompatibility, promoting wound healing and reducing inflammation.^43^

These findings collectively demonstrate that Tz/Nb click chemistry is a robust and versatile tool for modifying SF materials, making it a valuable strategy for developing advanced biomaterials with improved bioactivity and structural properties.^44^

### 3.3 Water Vapor Transmission Rate (WVTR)

Moisture balance is necessary for optimal wound healing. Moisture permeability, measured by water vapor transmission rate (WVTR), is an important parameter for wound dressings.^34^ Higher WVTR can lead to fast dehydration of the recovering tissue, forming larger scars. Meanwhile, lower WVTR might cause an accumulation of exuding fluid at the wound surface as well as the inflammation and maceration of the surrounding tissue.^9,45^ Moreover, hydrogels have high WVTRs; thus, they do not represent a barrier to vapor permeation and should moisturize the wound environment by delivering water molecules to the wound.^46^ In this study, the key factor for the WVTR of the hybrid material is the SF membrane since it forms a dense phase, limiting vapor permeation.

The values of water vapor permeability (WVP), water vapor transmission rate, and thickness for the silk fibroin membrane were 4.84 g·mm/m^2^·day·kPa, 283.11 g/m^2^.day, and 0.041 mm, respectively. The thickness and WVP are in the same range as in our previous study with SF/alginate blends.^29^ Recent studies on silk fibroin (SF)-based materials demonstrate a broad range of WVTR values depending on the composition and structure. For instance, hydrogel-coated SF fabrics exhibited WVTRs around 480 g/m^2^·day, aligning with requirements for moderate exudate management.^47^ Additionally, silk fibroin blended with poly­(vinyl alcohol) and copaiba oleoresin yielded WVTRs around 622.8 g/m^2^·day, highlighting the tunability of SF composites for moisture control in various biomedical applications.^48^ These results confirm that silk fibroin-based materials, particularly in hybrid formulations, can be engineered to meet the specific WVTR requirements for different wound types.

In previous studies, silk fibroin membranes were cross-linked using ethanol, a chemical treatment that is more aggressive than annealing and tends to increase membrane crystallinity, with a water vapor permeability of 2.7 g·mm/m^2^·day·kPa.^29^ In contrast, the higher WVP value observed in the present study may be attributed to the milder annealing treatment, which results in a less crystalline structure and consequently allows greater water vapor transmission.

Moreover, hydrogels are three-dimensional cross-linked polymer networks composed of hydrophilic polymers with high water contents. This structure allows for excellent moisture retention, breathability, and adaptability to the wound site, creating an optimal environment for tissue regeneration. Incorporating mucin into hydrogel formulations can significantly influence their WVP, a critical factor in wound healing applications. For instance, mucus-mimicking mucin-based hydrogels synthesized through tandem chemical and physical cross-linking exhibited water content ranging from 97.6 to 98.1%, closely resembling native mucus properties. These hydrogels demonstrated mechanical stability and permeability suitable for maintaining a moist wound environment conducive to healing.^49^ A recent study developed self-healing and antioxidant mucus-inspired hydrogels by dynamically cross-linking mucin with phenylboronic acid-functionalized polymers. These hydrogels not only showcased excellent moisture retention but also provided a protective barrier against oxidative stress, further enhancing their applicability in wound care.^50^ The incorporation of mucin into hydrogel systems thus offers a dual advantage: maintaining optimal hydration levels and providing a protective interface, both essential for effective wound healing.

To contextualize our findings, it is relevant to compare them with WVTR values reported for commercial and previously studied hydrogel-based dressings. Wu et al. evaluated the WVTR characteristics of some commercial hydrogels and found their WVTRs to be in the range of 76–9360 g/m^2^.day for Dermiflex (Johnson & Johnson) and Vigilon (Bard).^51^ Demeter et al. summarized scientific contributions on hydrogels produced by irradiation and found WVTR values ranging from 40 to 4600 g/m^2^.day for cross-linked PVA/chitosan blends and AgNP/gelatin/PVA, respectively.^52^

### 3.4 Epithelial Growth Factor (EGF) Release

Biomaterials can release compounds to enhance wound healing, such as epithelial growth factor (EGF), which can activate processes that improve dermal regeneration.^27,28^ Here, we investigated the efficiency of the SF membranes (prior and after tetrazine functionalization), mucin hydrogel in two concentrations, and the hybrid material for the incorporation and release of EGF.

The release profile of EGF from SF membranes is presented in Figure 4A. The release reached constant levels (plateau) after 8 days. Less than 40% of EGF was released, with more than half being released in the first 24 h, meaning a continued release of EGF, suggesting interaction between silk fibroin and EGF. This result can be interesting in cases where a slow release of the active is desired. The initial burst release of EGF, Figure 4B, is considered favorable because it helps to rapidly activate the keratinocytes.^53^

**Figure 4:**
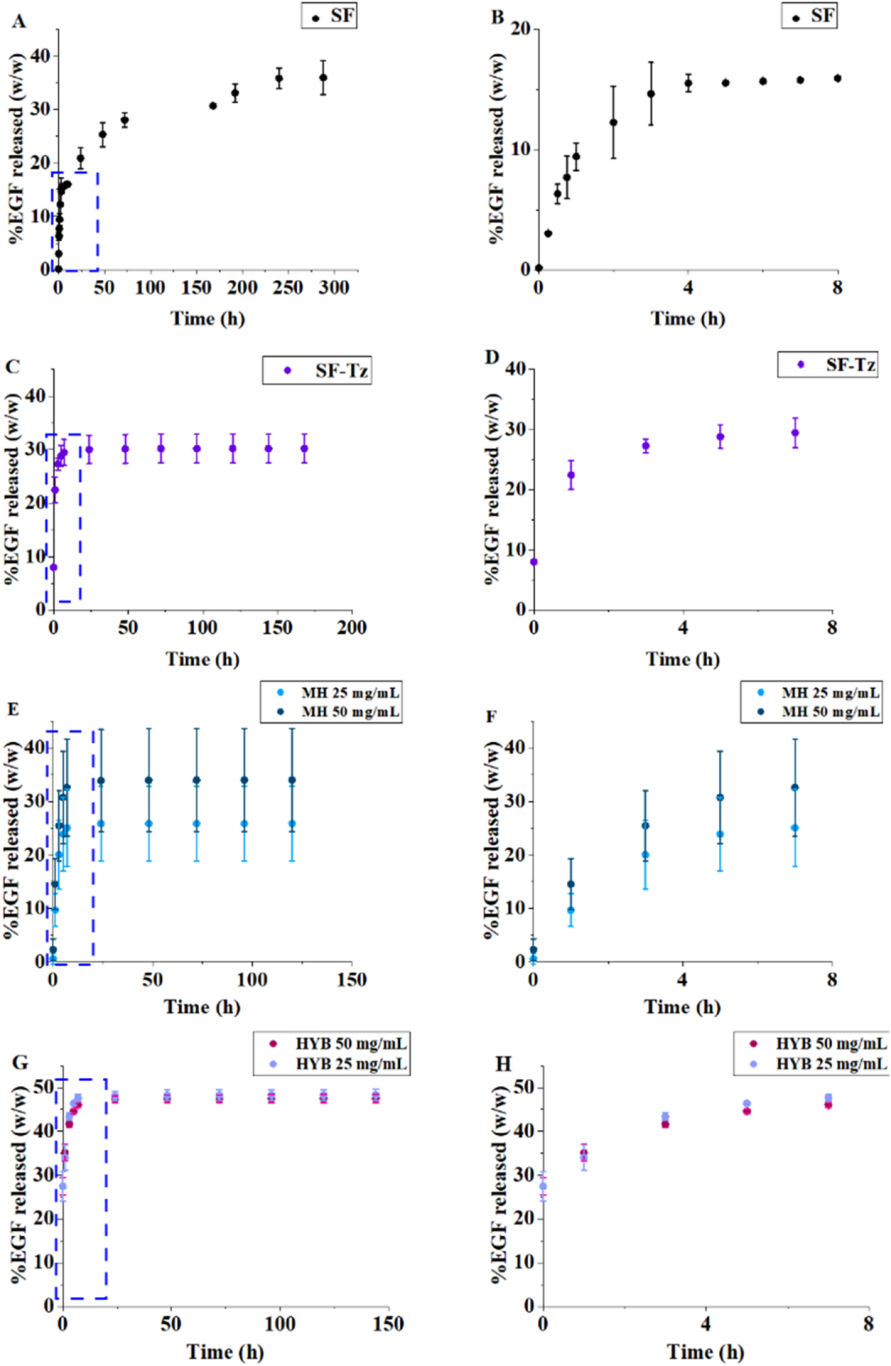
Graphs on the left represent the total release time, while graphs on the right represent a zoom on the initial release of up to 8 h. (A) Release profile of EGF in SF membranes. (B) EGF release in SF over the first 8 h. (C) EGF release profile from the SF-Tz membrane. (D) EGF release from the SF-Tz membrane over the first 8 h. (E) EGF release profile from MH with different concentrations of 25 and 50 mg/mL. (F) EGF release in MH over the first 8 h. (G) EGF release profile from HYB. (H) EGF release in HYB over the first 8 h.

The EGF release profile for the SF-Tz membrane is shown in Figure 4C. It can be seen that the percentage released is close to 30%, Figure 4D, with equilibrium being reached in about 24 h. The fact that only a fraction of the active was released suggested that there may be interactions between silk fibroin and the growth factor.

EGF was loaded into the mucin–tetrazine and mucin–norbornene solutions prior to hydrogel preparation. Figure 4E shows the EGF release profile for mucin hydrogels with different concentrations, 25 and 50 mg/mL. The system reaches equilibrium within the first 24 h, and there is no statistically significant difference between 25 and 50 mg/mL hydrogels, Figure 4F. According to the microscopy images of mucin hydrogels, it is possible to notice a slight difference in the size of the porous of each hydrogel: the pore diameters were 0.085 ± 0.029 μm and 0.147 ± 0.053 μm for the 25 and 50 mg/mL samples, respectively, Figure S1. Considering that both materials have the same concentrations of Tz and Nb, the higher amount of mucin leads to larger pores. However, this fact does not seem to influence the release kinetics because the release profile in both hydrogels is quite similar.

The EGF release profile of the hybrid material is shown in Figure 4G. EGF was incorporated into the functionalized SF before casting at room temperature. The results are the average and standard deviation from triplicate experiments. It was not possible to notice a significant difference between the membranes regarding the mucin concentration, suggesting that the release depends on silk fibroin. There is a slight difference in the release speed in the initial hours, Figure 4H, as a possible consequence of different porous sizes. However, it does not seem to be decisive in releasing EGF. The release profiles are similar to those of the SF-Tz membrane, reaching the plateau in the first 24 h. The HYB has released more EGF after 48 h (around 48%) than the SF membrane (16%), the SF-Tz membrane (30.17%), and MH (26 and 34% for 25 and 50 mg/mL, respectively), which points to a synergistic effect on the hybrid material, probably related to the interactions between the fibroin functionalized with tetrazine and the mucin hydrogel. The dense SF membrane and the porous network on the MH seem to provide a faster path for the EGF release. The high amount of EGF released in the first 24 h is an interesting feature since this growth factor acts in wound healing by the stimulation of the proliferation and migration of keratinocytes, a process that is more effective in the first 5 days after the injury. For instance, EGF application after this period produces no significant improvement over controls since by this time re-epithelialization has already occurred in both groups.^54^

The increase in the total EGF released by the hybrid material compared with SF materials from previous works is very interesting. Schneider et al. worked with silk mats containing EGF and observed a slow release in a time-dependent manner (25% EGF release in 170 h). When applied to full-thickness skin wounds in rats, these EGF-loaded mats significantly accelerated wound closure compared to controls, demonstrating enhanced re-epithelialization and granulation tissue formation.^54^ Chouhan et al. prepared nonmulberry SF-based electrospun mats and functionalized them with EGF, obtaining a maximum of 34.71% EGF release in 72 h. Their *in vivo* wound healing assessment demonstrated accelerated wound healing, enhanced re-epithelialization, highly vascularized granulation tissue, and higher wound maturity.^55^ Biomaterial-based delivery systems for EGF have demonstrated notable benefits in promoting cell proliferation and tissue regeneration during wound healing. For instance, EGF was incorporated into alginate-based hydrogels cross-linked with heparin to achieve controlled release. The hydrogels released EGF over a period of 5 days. Application of these EGF-loaded hydrogels to full-thickness skin wounds in rats resulted in accelerated wound closure, enhanced re-epithelialization, and increased collagen deposition compared to controls. Histological analysis confirmed improved tissue regeneration in the treatment group.^56^

The EGF release study was also used for mathematical modeling. The results from the Korsmeyer–Peppas model analysis are presented in Table S2. For the release of EGF from the silk fibroin membrane, Figure 4A, the parameter values are *k* = 10.41 (min^–*n*^) and *n* = 0.22 with *R* ^2^ = 0.98. The *n* value indicates that the Fickian diffusion controls the mass transfer process. The *n* value lies below 0.5; therefore, the complex Fickian diffusion is the mechanism of drug release, which consists of drug diffusion through the swollen hydrogel and/or water-filled pores.^57^ This mechanism was also observed for the release of diclofenac sodium from silk fibroin.^58^

### 3.5 Papain Release

In addition to the epithelial growth factor, the silk fibroin/mucin hybrid material was also tested for the incorporation and release of papain. This protein is widely studied on wound healing due to its anti-inflammatory and debridement features.^24,59^ In this study, papain solution was loaded on the SF-Tz membrane and the hybrid material, Figure 5. Given the comparable release profiles observed for mucin hydrogels at 25 and 50 mg/mL, the 25 mg/mL formulation was selected for both the papain release study and the cell viability assay, as it offers similar performance while minimizing material usage.

**Figure 5:**
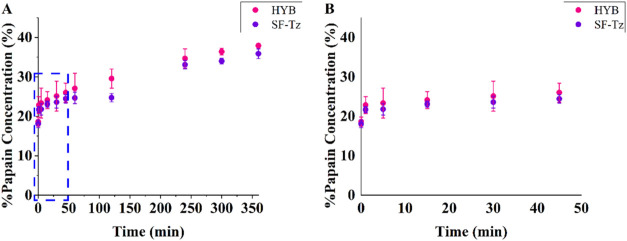
(A) *In vitro* release profiles of papain from the SF-Tz membrane and the hybrid material: tests were performed in triplicate. (B) Inset.

Although the release profiles of both the hybrid material and the SF-Tz membrane were similar, the later was slower; especially between 60 and 180 min time points. Similar values of the initial burst release of papain were observed within the first 60 min. After that, the release was fast, and both the SF-Tz membrane and the hybrid material had a similar cumulative release, up to 27%. Afterward, while the hybrid material reached a cumulative release of 30% for 180 min, the SF-Tz membrane had around 25% of release and showed sustained drug release behavior. Results indicate that the mucin hydrogel layer on the hybrid material accelerated the release. From this point on, both materials reached approximately 36% at 360 min.

The release profile, with an initial burst release (Figure 5B), is in accordance with previous works, reporting papain release from different materials, Table 1. Researchers developed alginate-based wound dressings incorporating papain to enhance therapeutic features. The study optimized conditions for papain immobilization and assessed enzyme activity stability over 28 days. *In vitro* cytotoxicity assays using fibroblasts and keratinocytes indicated that the bioactive material maintained proteolytic properties and was nontoxic, suggesting its potential as an effective wound dressing.^23^ A representative study demonstrating the therapeutic benefits of papain-loaded biomaterials in wound healing involved a hydrogel composed of poly­(γ-glutamic acid), chitooligosaccharide, and papain, aiming to prevent hypertrophic scar formation during skin wound healing. Applied to a rabbit ear skin wound model, as an effect of the introduction of papain, the hydrogel inhibited excessive collagen deposition and the generation of hyperplastic scars effectively.^60^

**Table 1:** Literature Review on Papain Release

author	material	papain released (%)	time (h)
this study	silk fibroin/mucin hydrogel hybrid	36	6
Shoba et al.^61^	poly(vinyl alcohol) (PVA) nanofibers	∼45	6
		55	24
Moreira Filho et al.^23^	Ca alginate membrane	∼20	6
		64.1	24
Lima et al.^62^	carboxymethylcellulose and PVA-based gel	∼48	48
		82	96
Vasconcelos et al.^63^	oxidized bacterial cellulose	26.55	6
		88.5	72

The kinetic release parameters and regression coefficients from the Korsmeyer–Peppas equation are presented in Table S3. For the release of papain from the hybrid material (Figure 5), *k* is 17.20 min^–n^, with *R* ^2^ = 0.89. The *n* value is 0.12, indicating complex Fickian diffusion, as in the case of EGF release.

### 3.6 Cell Viability

Cell viability is an indicator of biocompatibility used to demonstrate that the materials are suitable for wound healing. The results of the cell viability assay with HaCat cells using the AlamarBlue assay are shown in Figure 6. Previous works have shown the noncytotoxicity of materials produced from silk fibroin, Table 2. According to ISO 10993-5, cell viability percentages over 80% are classified as noncytotoxic; those between 80 and 60% as weakly cytotoxic; those between 60 and 40% as moderately cytotoxic; and those below 40% highly cytotoxic.^64^ Since the materials were placed on top of the cell culture, the reduction in the number of viable cells may also be attributed to the effect of the material on adhesion and its potential to block cell growth.

**Figure 6:**
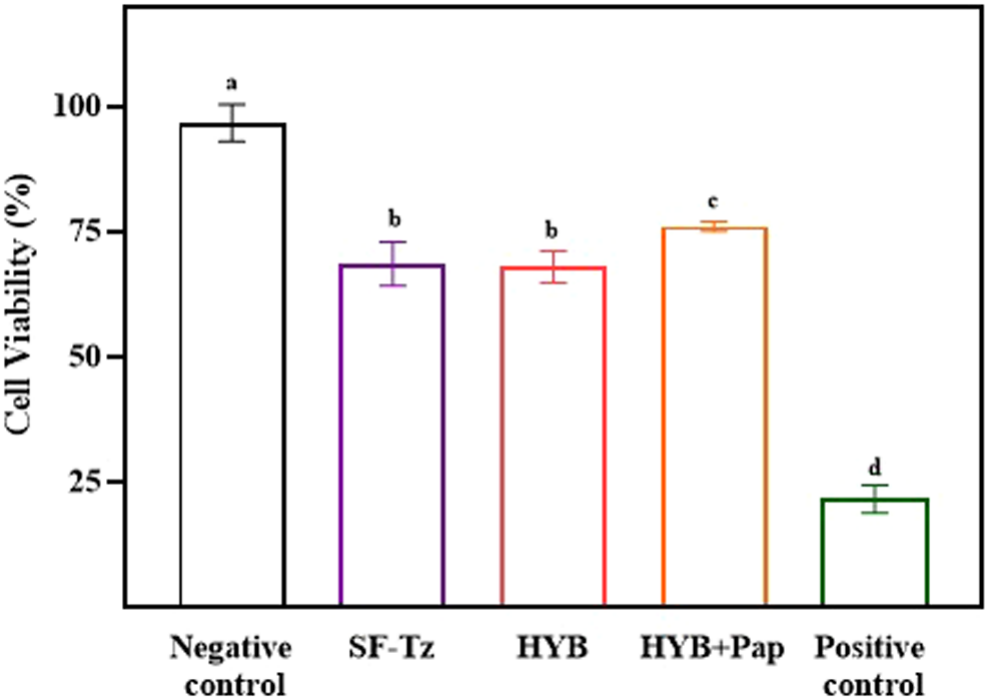
Cell viability of human keratinocytes (HaCat) determined by the AlamarBlue assay for the SF membrane with tetrazine grafting and the hybrid material with and without papain immobilization. ^a,b,c^ Different letters in the same column indicate statistically significant differences (*p* < 0.05) between the means, according to Tukey’s test.

**Table 2:** Cell Viability of Materials Containing Silk Fibroin (SF)

author	material	assay	cell line	cell viability
this study	SF-Tz	AlamarBlue	HaCat	66.8% (24 h)
	HYB			68% (24 h)
	HYB + Papain			76% (24 h)
Keihan et al.^65^	SF/chitosan hydrogel/Mg(OH)_2_	MTT	Hu02	62.5% (72 h)
Ameer et al.^66^	SF	MTT	L929	100%
Karahaliloğlu^67^	SF/curcumin	MTT	HaCat	SF: 94%
				SF/0.005 curcumin: 96%
				SF/0.01 curcumin: 90%
Karahaliloğlu^67^	SF/curcumin	MTT	L929	SF: 89%
				SF/0.005 curcumin: 83%
				SF/0.01 curcumin: 81%

The hybrid material and the hybrid with immobilized papain showed cell viabilities for HaCat of 68% (±3.27) and 76% (±1.07), respectively, Figure 6. The hybrid material with papain immobilization (HYB + Pap) shows a marked improvement in cell viability compared to SF-Tz and HYB, likely due to the bioactive role of papain in enhancing the microenvironment suitability for cell proliferation. However, the proteolytic nature of papain may have contributed to a reduction in biocompatibility compared to the control by degrading the extracellular matrix secreted by the cells and disrupting glycoprotein adhesion, causing cell detachment from the well bottom.^59^ Despite these effects, the materials developed in this study can still be considered suitable for wound dressing applications. The results of the cell viability of the hybrid material are also compatible with the current literature, as shown in Table 2.

Regarding the biocompatibility of the mucin hydrogel, previous works point to the noncytotoxicity of this material. Yan et al. investigated whether mucin hydrogels could provide a biocompatible microenvironment for cells and microtissues to be transplanted. MIN6m9 cells were incubated in mucin hydrogels with 10% AlamarBlue in a complete cell culture medium for 4 h in a humidified incubator (37 °C, 5% CO_2_) on days 1, 4, 6, 11, 22, and 28.^68^ The fluorescence method was used to measure cellular metabolic activity under conditions similar to this work. The authors reported that islet-like cells or organoids continuously increased in metabolic activity after day 4, as measured by the AlamarBlue assay, not exhibiting any signs of necrosis. Furthermore, there was no sign that the hydrogel degraded throughout the experiment.

## 4 Conclusions

This study successfully developed a hybrid material combining a silk fibroin (SF) membrane with a mucin hydrogel (MH), utilizing functionalization with tetrazine and norbornene. The hybrid material was achieved through click chemistry, enabling its precise assembly. Morphological analyses revealed a dense silk fibroin phase alongside a porous mucin gel phase, while physical characterization indicated compliance with standards for commercially available dressings. The hybrid material demonstrated effective drug-hosting capabilities, successfully incorporating bioactive agents such as epidermal growth factor (EGF) and papain. Notably, a synergistic interaction between the SF and mucin hydrogel components was observed, leading to enhanced EGF release from the hybrid material compared to standard SF-based dressings. Moreover, the material exhibited sustained papain release while maintaining favorable cell viability, underscoring its potential as a versatile wound dressing. Further evaluation of the antioxidant and antibacterial properties of the hybrid material is necessary to strengthen its applicability as a multifunctional wound dressing.

## Supplementary Material



## Notes

Main bands observed by infrared spectrum of the silk fibroin membrane, mucin hydrogel and material loaded with EGF, pore labeling for MH images at difference concentrations and parameters of the Korsmeyer–Peppas model for the EGF and Papain release (Appendix A) (PDF)
