# Supplementary material for: Functionalized Silk Fibroin and Mucin Hybrid Material for Targeted EGF and Papain Delivery in Wound Healing
Source: ACS Omega. 2025 Aug 13;10(33):37432–44. doi: 10.1021/acsomega.5c03320 (PMC12391997; doi:10.1021/acsomega.5c03320)
Supplement: Supplementary file 1 [file ao5c03320_si_001.pdf]

Electronic Supplementary Information

Functionalized Silk Fibroin and Mucin Hybrid Material for Targeted EGF and Papain Delivery in Wound Healing

Fernando José Soares Barros[1*]; Laise Maia Lopes[1,2*]; Sedef Ilk[1,3]; Rodrigo Silveira Vieira[4]; Thomas Crouzier[1,5]; Mariana Agostini de Moraes[2,6;] Marisa Masumi Beppu[2**]

[1] *KTH Royal Institute of Technology, Department of Chemistry, Division of Glycoscience, SE-10044, Stockholm, Stockholm County, Sweden*

[2] *State University of Campinas (UNICAMP), School of Chemical Engineering, Department of Materials and Bioprocess Engineering, Av Albert Einstein, 500, CEP 13083-852, Campinas, SP, Brazil*

[3] *Niğde Ömer Halisdemir University, Faculty of Medicine, Department of Immunology, TR-51240, Niğde, Turkey*

[4] *Federal University of Ceará (UFC), Department of Chemical Engineering, Campus do Pici, Bloco 709, CEP 60440-900, Fortaleza, CE, Brazil*

[5] *Technical University of Denmark (DTU), Department of Health Technology, Ørsteds Plads, Building 345C, DK-2800, Lyngby, Denmark*

[6] *Federal University of São Paulo (UNIFESP), Department of Chemical Engineering, Rua São Nicolau, 210, CEP 09913-030, Diadema, SP, Brazil*

* Fernando José Soares Barros and Laise Maia Lopes contributed equally to this work

**Corresponding Author:

Marisa Masumi Beppu - beppu@unicamp.br

Table S1: Summary of the main bands observed by infrared spectrum (FTIR-ATR) of the SF membrane, SF-Tz membrane, SF-Tz membrane loaded with EGF, mucin hydrogel (MH) and MH loaded with EGF.

**Material**	**Wavenumber (cm^{-1})**					
MH	1726	1643	1544	1443	1245	1038
MH-EGF	1726	1662	1551	1444	1245	1077
SF	-	1643	1626	1532	1238	-
SF-Tz	-	1681	1630	1566	1238	-
SF-Tz-EGF	-	1651	1547	1536	1242	-

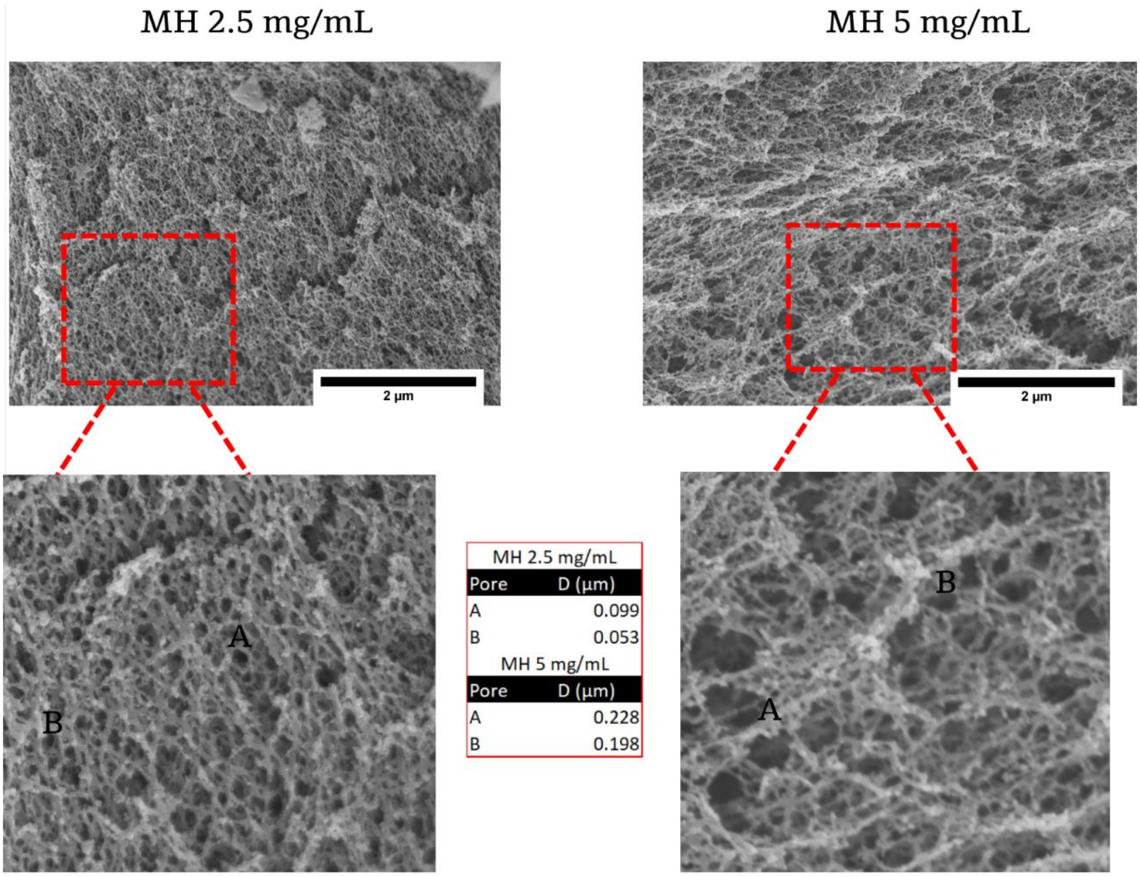

Figure S1: Examples of pore labeling for MH with different concentrations – 25 mg/mL and 50 mg/mL.

The average pore diameter was determined by analyzing 50 pores per image using ImageJ software. For illustrative purposes, the figure highlights two representative pores for each mucin hydrogel concentration to demonstrate typical pore size and morphology.

Table S2. Parameters of the Korsmeyer-Peppas model for the EGF release from the SF membrane, SF-Tz membrane, MH and hybrid material.

**Material**	**Korsmeyer-Peppas Model**		
	R^2	k (min^{-n})	n
HYB 5%	0.752	42.413	0.026
HYB 2.5%	0.630	44.412	0.019
MH2.5%	0.513	17.163	0.101
MH5%	0.619	26.983	0.055
SF	0.981	10.409	0.221
SF-Tz	0.718	27.746	0.017

Table S3. Parameters of the Korsmeyer-Peppas model for the papain release from the SF-Tz membrane and hybrid material.

**Material**	**Korsmeyer-Peppas Model**		
	**R^2**	**k (min^{-n})**	**n**
SF-Tz	0.696	16.846	0.107
HYB	0.890	17.203	0.125
